# A systematic review of implementation frameworks of innovations in healthcare and resulting generic implementation framework

**DOI:** 10.1186/s12961-015-0005-z

**Published:** 2015-03-14

**Authors:** Joanna C Moullin, Daniel Sabater-Hernández, Fernando Fernandez-Llimos, Shalom I Benrimoj

**Affiliations:** Graduate School of Health, Pharmacy, University of Technology Sydney, Broadway, PO Box 123, Ultimo, 2007 NSW Australia; Academic Centre in Pharmaceutical Care, Pharmaceutical Care Research Group, Faculty of Pharmacy, University of Granada, 18071 Granada, Spain; Institute for Medicines Research (iMed.UL), Department of Social Pharmacy, Faculty of Pharmacy, University of Lisbon, Avda. Prof. Gama Pinto, 1649-019 Lisbon, Portugal

**Keywords:** Diffusion, Framework, Implementation, Knowledge translation, Model, Systematic literature review, Theory

## Abstract

**Background:**

Implementation science and knowledge translation have developed across multiple disciplines with the common aim of bringing innovations to practice. Numerous implementation frameworks, models, and theories have been developed to target a diverse array of innovations. As such, it is plausible that not all frameworks include the full range of concepts now thought to be involved in implementation. Users face the decision of selecting a single or combining multiple implementation frameworks. To aid this decision, the aim of this review was to assess the comprehensiveness of existing frameworks.

**Methods:**

A systematic search was undertaken in PubMed to identify implementation frameworks of innovations in healthcare published from 2004 to May 2013. Additionally, titles and abstracts from *Implementation Science* journal and references from identified papers were reviewed. The orientation, type, and presence of stages and domains, along with the degree of inclusion and depth of analysis of factors, strategies, and evaluations of implementation of included frameworks were analysed.

**Results:**

Frameworks were assessed individually and grouped according to their targeted innovation. Frameworks for particular innovations had similar settings, end-users, and ‘type’ (descriptive, prescriptive, explanatory, or predictive). On the whole, frameworks were descriptive and explanatory more often than prescriptive and predictive. A small number of the reviewed frameworks covered an implementation concept(s) in detail, however, overall, there was limited degree and depth of analysis of implementation concepts. The core implementation concepts across the frameworks were collated to form a Generic Implementation Framework, which includes the process of implementation (often portrayed as a series of stages and/or steps), the innovation to be implemented, the context in which the implementation is to occur (divided into a range of domains), and influencing factors, strategies, and evaluations.

**Conclusions:**

The selection of implementation framework(s) should be based not solely on the healthcare innovation to be implemented, but include other aspects of the framework’s orientation, e.g., the setting and end-user, as well as the degree of inclusion and depth of analysis of the implementation concepts. The resulting generic structure provides researchers, policy-makers, health administrators, and practitioners a base that can be used as guidance for their implementation efforts.

**Electronic supplementary material:**

The online version of this article (doi:10.1186/s12961-015-0005-z) contains supplementary material, which is available to authorized users.

## Background

Implementation of innovations into practice is a complex process [1]. The importance and acceptance of implementation research is growing and as a result is rapidly evolving [2]. The fields of implementation science and knowledge translation have arisen to drive change and move an array of innovations into practice [3-5].

Implementation and knowledge translation frameworks have predominantly developed within disciplines. This discipline-specific approach in the targeted innovations, settings, and end-users, has resulted in multiple and potentially disparate frameworks being developed and used [4,6-12]. Variations in implementation frameworks include the presence of disparate terminology and classification of implementation concepts. Implementation concepts are designated as including the process of implementation (divided into a series of stages or steps), domains (groups or levels of influences), and three elements: factors (also called barriers and enablers or determinants of practice), strategies (approaches to address the factors and implement the innovation), and evaluations. As implementation science advances, researchers have attempted to consolidate nomenclature and develop multidisciplinary frameworks [13-15]. Yet, it is unknown to what extent frameworks continue to focus on one concept alone or be innovation-specific.

Selecting an implementation framework is a challenging task. If an organisation or provider is interested in implementing a particular innovation, they must decide if an implementation framework for the innovation to be implemented is the most suitable, or should a framework or combination of frameworks, potentially created for the implementation of different innovation(s), be considered? In other words, as implementation frameworks vary in their orientation, it is plausible, by design or otherwise, that not all frameworks targeting a particular innovation cover all implementation concepts. The diversity of frameworks leads to a second question: do implementation frameworks across the range of innovations in healthcare consist of the same concepts, covered to the same degree and depth, and if not, how do they vary? Core implementation concepts have been posited and so it would appear for those with an objective to implement an innovation, rather than, for example, study a particular concept, the consequences of using a framework lacking degree or depth of an implementation concept may be poor results [9]. Therefore, the answers to such questions of framework comprehensiveness may aid users in their selection of a suitable implementation framework or whether to combine multiple implementation frameworks to aid their implementation efforts.

In 2004, Greenhalgh *et al.* [16] conducted a comprehensive literature review of implementation studies for innovations in service delivery and organisation. The work was focused predominantly in healthcare and used a snowballing technique to locate studies, as formal search techniques at this time drew a poor yield. Their landmark review located and analysed research areas that provided evidence of implementation research, in addition to collating findings to create a conceptual framework for implementation. The review elicited attributes of innovations, receiving organisations and their surrounding contexts; the complex, stop-start nature of the implementation process (from diffusion and dissemination, to adoption/assimilation and implementation/routinisation); as well as positing preliminary links amongst implementation concepts. In the ensuing 9 years, the field has expanded considerably and further taxonomies, checklists, conceptual frameworks, theories, and models of implementation have been developed [10,13,14,17,18]. A number of literature reviews of implementation frameworks have also been conducted, concentrating on particular implementation concepts, such as a particular stage, or specifically on either the factors, strategies or evaluations, rather than addressing all the concepts that could affect an innovation’s implementation [13,17,19-26]. There seems to be no literature review covering the comprehensiveness of the frameworks [26].

With the expansion of implementation literature and maturation of the implementation field, it is now possible to conduct a formal search strategy solely within healthcare. The focussed results will increase the study’s relevance and applicability to those comparing and selecting implementation frameworks for innovations in healthcare. It therefore appears timely to conduct a systematic review to analyse the comprehensiveness of implementation frameworks of innovations in healthcare. The present systematic review aimed to identify the extent to which existing implementation frameworks include core implementation concepts and determine if frameworks vary depending on the innovation they target.

## Methods

### Search strategy

A systematic literature search was undertaken to identify all frameworks of implementation of innovations in healthcare published from 2004 to May 2013. A search of literature was conducted using PubMed without language restrictions. The search strategy used was: (“Models, Educational” [MH] OR “Models, Nursing” [MH] OR “Models, Organizational” [MH] OR “Models, Psychological” [MH]) AND (“Diffusion of Innovation” [MH] OR “Organizational Innovation”[MH] OR “Capacity Building” [MH] OR “Decision Making, Organizational” [MH] OR “Organizational Culture” [MH] OR “Information Dissemination” [MH]) AND has abstract AND (model [TIAB] OR models [TIAB] OR theory [TIAB] OR theories [TIAB] OR framework* [TIAB]). In addition, titles and abstracts of all *Implementation Science* journal articles (Feb 2006 to May 2013) and references from identified papers were reviewed for implementation frameworks.

### Inclusion/exclusion criteria

Papers were included if they proposed an implementation framework of an innovation in healthcare. The inclusion criteria were defined as follows (Additional file 1):

- *Implementation* was defined as the process of putting to use or integrating innovations within a setting [14]. Frameworks needed to include concepts related to the either the stage of ‘operation’ (where the innovation is in use and is in the process of being integrated into routine practice) and/or ‘sustainability’ (the process of maintaining innovation use, capacity and benefits).
- *Framework* was defined as a graphical or narrative representation of the key factors, concepts, or variables to explain the phenomenon of implementation [27-30], and as a minimum needed to include the steps or strategies for implementation. Papers were included if they proposed a framework, model, or theory of implementation. Eligible papers needed to describe a new, or make change(s) to an existing, implementation framework.
- *Innovation in healthcare* was defined as a novel idea or set of behaviours, routines, and/or ways of working that involve a change in practice within a healthcare setting [6,16].

Frameworks were excluded if they were:

- Focussed on one specific domain, factor, or strategy (for example, organisational context, climate, or behavioural change).
- Studies applying or validating a framework without proposing a change to the framework.
- Based on a single case study.
- Quality improvement frameworks.
- For the implementation of a culture (for example, safety culture or green culture within an organisation).
- A model of patient care.
- To develop the fields of implementation science and knowledge translation (for example, the training of students in implementation).
- Concentrating on collaborative education as a method for change and models for curricula reform.

### Data collection

A single reviewer (JCM) assessed titles and abstracts. For those that appeared to meet the inclusion criteria, the full paper was obtained and assessed. Any papers the reviewer was unsure about were discussed with a second member of the research team (SIB) and agreed upon for inclusion or exclusion.

### Data extraction

The literature was critically analysed, by the same reviewer (JCM), to evaluate the frameworks according to the definitions provided and subsequently extract the following features from the frameworks:

i. The orientation of the framework: the kind of innovation (as described by the authors), the healthcare setting in which the innovation was to be implemented, the planned end-user(s), and a summary of the overall aim for which the framework was developed.
ii. The type of the framework: descriptive, prescriptive, explanatory, or predictive [31,32].
  Descriptive frameworks describe the properties, characteristics, and/or qualities of implementation.
  Prescriptive frameworks provide direction on the implementation process via a series of steps or procedures.
  Explanatory frameworks specify the linkage and/or relationships between framework concepts.
  Predictive frameworks hypothesise or propose directional relationships between the concepts of implementation.
iii. The implementation stages covered by the framework. Stages were designated based on Greenhalgh et al. conceptual framework (diffusion and dissemination, adoption/assimilation, and implementation) [16]. In addition, the pre-implementation stage of ‘development’ (innovation creation, refinement, and impact evaluation) from knowledge translation [12], and post-implementation stage of ‘sustainability’, which had not been included in the review by Greenhalgh et al. [16] due to lack of studies at that time focussing on this stage, were added. Diffusion and dissemination were combined under the heading of ‘communication’ (process by which people share information about a new innovation to increase awareness) as the terms often appear concurrently. The adoption/assimilation phase was divided into two sub-stages of ‘exploration’ (the innovation-decision process, whereby the end-user(s) appraise the innovation to decide whether to adopt) and ‘installation’ (the course of preparation, prior to use) [33-35]. The final stages included in the review table for analysis were development, communication, exploration, installation, operation, and sustainability. A framework was marked as including a stage if process components fitted the definitions of stage as provided in Additional file 1.
iv. The domains addressed in the framework. The domains were based on the Consolidated Framework for Implementation Research (intervention characteristics, outer setting, inner setting, characteristics of individuals, and process) [13]. The outer setting was divided into two, the ‘external system’ (economic, political, and professional milieu) and ‘local environment’ (circumstances surrounding the organisation(s) including patient, community, network) as it has been suggested the local environment has been lacking emphasis in previous frameworks [36,37]. In addition, the inner setting was termed ‘organisation’ and intervention called ‘innovation’ for greater clarity. The process factors were included under the ‘strategies element’ rather than as a domain. The final domains included in the review table for analysis were innovation, individuals, organisation, local environment, and external system. A framework was marked as including a domain if the influences fitted the definitions as provided in Additional file 1.
v. a) The degree of inclusion of the three elements: influencing factors, strategies, and evaluations (Additional file 1 for definitions), coded based on the substantiation provided for their inclusion. That is, where a smaller range of factors, strategies, and/or evaluations were provided, not a comprehensive range, the article was classified based on the justification of inclusion rather than number. These were assessed through classification into three levels:
  + The framework itemises a range of factors, strategies, or evaluations with no explanation for their inclusion;
  ++ The framework itemises a range of factors, strategies, or evaluations with some form of justification for their inclusion;
  +++ The framework itemises a comprehensive range of factors or strategies based on a literature review or evaluations covering each of the concepts included in the framework.
b) The depth of analysis of the three elements: influencing factors, strategies, and evaluations. These were assessed through classification into three levels:
  ^ Factors, strategies, or evaluations provided as a list without descriptions;
  ^^ Factors, strategies, or evaluations provided with descriptions;
  ^^^ Factors, strategies, or evaluations provided with descriptions which included the relationships between or within the elements (factors, strategies, and evaluations) or mechanisms for operationalization.

### Synthesis of results

A table was constructed to incorporate all of the data extracted (Additional file 2). Frameworks were ordered based on the innovation for which the framework was orientated and, secondly, on the setting. The classification of innovations into groups was based on the terminology used in the articles rather than by *ad hoc* definitions.

## Results

**Figure 1:**
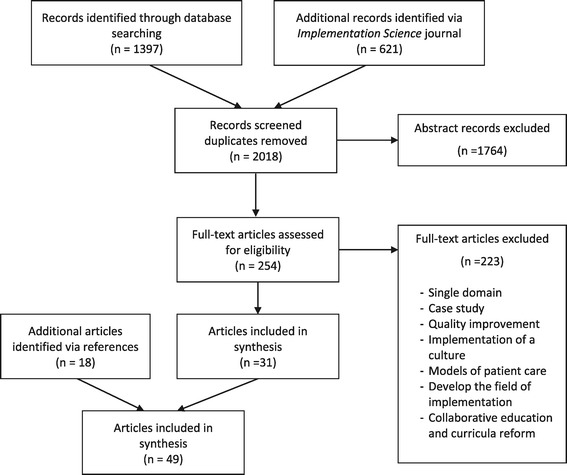
**PRISMA flow chart of framework selection [** 38 **].**

The database search identified 1,397 articles and a further 621 were sourced from *Implementation Science* journal. From the 2,018 articles screened, 1,764 articles were eliminated after title and abstract screening and a further 223 after examination of the full-text articles. The references of the remaining 31 articles were screened, resulting in the identification of an additional 18 frameworks. Finally, a total of 49 implementation frameworks of an innovation in healthcare were included in the systematic analysis (Figure 1).

Frameworks were synthesised into tabular format (Additional file 2). Innovations were classified into groups: interventions (including those termed interventions, programs, innovations, complex interventions/innovations, shared-decision making, technologies, evidence-based practices, and telehealth; n = 22) [13,15,16,29,33-37,39-56], guidelines (including clinical-practice, best-practice, and evidence-based guidelines; n = 4) [57-61], knowledge (including knowledge, evidence, and research; n = 15) [12,62-68], evidence-based practice model (EBP model; n = 5) [69-74], and packaged implementation programs for innovations (n = 3) [75-77]. For implementation frameworks of ‘EBP model’ and ‘packaged implementation programs’, the innovation to be implemented is the model or program itself, which when implemented allows for the implementation of further innovations. Examples of the different types of innovations within each group as per the corresponding articles are provided in Additional file 2.

In many cases, within the innovation groups the frameworks’ settings were similar (Additional file 2). Guidelines, knowledge, and EBP model frameworks were largely for clinical practice settings, while implementation programs were for community, public health, or human service settings. Interventions could be divided into two sub-groups; 12 were in community settings and 10 in clinical settings.

Key variations were seen between the innovation groups and the corresponding framework ‘types’ (descriptive, prescriptive, explanatory, and predictive) (Table 1). The ‘type’ of implementation framework for the innovation groups of interventions, guidelines, and implementation programs were often descriptive, in comparison to frameworks for knowledge and EBP model. Prescriptive frameworks, whereby the steps involved in the process of implementation were detailed, were rarely found for frameworks to implement guidelines or interventions, but were prevalent for implementation programs, EBP model, and knowledge frameworks. Overall, there were a larger number of descriptive and explanatory frameworks compared to prescriptive and predictive.

**Table 1:** **Framework types**

**Innovation group**	**Type of framework**			
	**Descriptive**	**Prescriptive**	**Explanatory**	**Predictive**
Interventions (n = 22)	16 (73%)	1 (5%)	14 (64%)	7 (32%)
Guidelines (n = 4)	3 (75%)	–	–	1 (25%)
Knowledge (n = 15)	4 (27%)	7 (47%)	8 (53%)	–
Evidence-based practice model (n = 5)	1 (20%)	3 (60%)	1 (20%)	1 (20%)
Implementation programs (n = 3)	3 (100%)	2 (67%)	1 (33%)	–
**TOTAL (n = 49)**	**27 (55%)**	**13 (24%)**	**24 (49%)**	**9 (18%)**

Percentages were calculated using the total number of frameworks at each innovation group in the denominator. Percentages are not accumulative because each framework could be fit into multiple ‘type’ categories.

The process of implementation was depicted in various forms, including an array of linear, non-linear, recursive, or reiterative series of processes, steps, stages, or phases. The breakdown, categorisation, nomenclature, and order of the stages also varied. For example, eight frameworks did not explicitly mention stages [13,15,42,44,46,49,50,64,74], and the terminology ranged from ‘orientation, insight, acceptance, change, and maintenance’ [52], to ‘implement, assess, adopt, disseminate, integrate, implement, maintain’ [73] or ‘unfreezing, moving, refreezing’ [72]. Additional stages included innovation (in this situation meaning adaptation or reinvention) [34] and pilot testing [58].

The stage of operation (implementation) was found in all but three frameworks (94%), which were focused solely on sustainability. The pre-implementation stages of innovation development and communication were included in 24% and 37% of frameworks, respectively. The exploration stage was reported in 45% of frameworks and both the installation and sustainability stages were included in 63% of frameworks (Table 2).

**Table 2:** **Framework stage analysis by innovation groups**

**Innovation group**	**Stages**					
	**Development**	**Communication**	**Exploration**	**Installation**	**Operation**	**Sustainability**
Interventions (n = 22)	3 (14%)	6 (27%)	9 (41%)	13 (59%)	19 (86%)	17 (77%)
Guidelines (n = 4)	1 (25%)	3 (75%)	1 (25%)	1 (25%)	4 (100%)	2 (50%)
Knowledge (n = 15)	6 (40%)	8 (53%)	8 (53%)	9 (60%)	15 (100%)	7 (47%)
Evidence-based practice model (n = 5)	1 (20%)	0 (0%)	2 (40%)	5 (100%)	5 (100%)	2 (40%)
Implementation programs (n = 3)	1 (33%)	1 (33%)	2 (67%)	3 (100%)	3 (100%)	3 (100%)
**TOTAL (n = 49)**	**12 (24%)**	**18 (37%)**	**22 (45%)**	**31 (63%)**	**49 (94%)**	**31 (63%)**

Percentages calculated as the number of frameworks (which included a stage or domain) divided by the number of frameworks in each innovation group.

When looking at the innovation groups, frameworks for the implementation of implementation programs covered the largest number of stages. Frameworks for the implementation of guidelines and knowledge included the communication stage more often than for those for implementation programs, interventions, and EBP model (75% and 53% vs. 33%, 27%, and 0%, respectively). In contrast, frameworks for implementation programs and interventions incorporated sustainability more frequently (100% and 77%, compared to 50% for guidelines, 47% for knowledge, and 40% for EBP model frameworks). Frameworks for the implementation of knowledge included the development stage most frequently (40% of frameworks), while frameworks for guidelines focussed largely on communication and operation only (Table 2).

The categorisation and explicit presence of domains also differed across the frameworks. For example, May’s [49] framework described only two domains (agency and context), and Aaron *et al.*’s [33] framework detailed three domains (outer context, inner context, and innovation); however, the components within the framework fitted four domains as per the definitions of analysis provided in Additional file 1. Three frameworks did not explicitly mention domains at all [35,54,56].

The organisational domain was covered most frequently in the 88% of frameworks, followed by the characteristics of the individuals involved in the process (76%), the innovation itself (73%), the local environment surrounding the implementation (55%), and the external system (45%). Frameworks for the implementation of implementation programs included all domains most often, followed by frameworks for interventions. Implementation frameworks for EBP model focussed largely on the individual and organisational domains, whereas frameworks for guidelines were more directed towards the guideline, or innovation itself, and the characteristics of the individuals (Table 3).

**Table 3:** **Framework domain analysis by innovation groups**

**Innovation group**	**Domains**				
	**Innovation**	**Individuals**	**Organisation**	**Local environment**	**External system**
Interventions (n = 22)	15 (68%)	15 (68%)	21 (95%)	14 (64%)	12 (55%)
Guidelines (n = 4)	4 (100%)	3 (75%)	2 (50%)	2 (50%)	2 (50%)
Knowledge (n = 15)	12 (80%)	11 (73%)	13 (87%)	7 (47%)	5 (33%)
Evidence-based practice model (n = 5)	2 (40%)	5 (100%)	4 (80%)	1 (20%)	1 (20%)
Implementation programs (n = 3)	3 (100%)	3 (100%)	3 (100%)	3 (100%)	2 (67%)
**TOTAL (n = 49)**	**36 (73%)**	**37 (76%)**	**43 (88%)**	**27 (55%)**	**22 (45%)**

Percentages calculated as the number of frameworks (which included a stage or domain) divided by the number of frameworks in each innovation group.

Out of the 49 implementation frameworks five comprehensively included the range of items within any one element with justification for their inclusion (as indicated by +++) and provided descriptions which included the relationships between or within the elements or mechanisms for operationalization (as indicated by ^^^) (Table 4). These frameworks were Damschroder *et al.* [13] covering factors, Kilbourne *et al.* [76] and Stetler *et al.* [55] for strategies, and Stetler *et al.* [55] and Lehman *et al.* [53] on evaluations. In total, only 6% of the frameworks covered the degree of any one element comprehensively (+++), while 20% covered factors, 29% strategies, and 14% evaluations in depth (^^^) (Table 4).

**Table 4:** **Framework element analysis (degree and depth)**

			**Factors (n = 49)**	**Strategies (n = 49)**	**Evaluations (n = 49)**
**Degree**	+++		3	3	3
	++		28	30	15
	+		17	16	18
	nil		1	–	13
**Depth**	^^^		10	14	7
	^^		19	22	13
	^		19	13	16
	nil		1	–	13
**Combined**	+++	^^^	1	2	2
	++	^^^	8	10	3
	+	^^^	1	2	2
	+++	^^	2	1	1
	++	^^	14	18	8
	+	^^	3	3	4
	+++	^	–	–	–
	++	^	6	2	4
	+	^	13	11	12
	nil		1	–	13

+ Degree and substantiation of inclusion; ^ Depth of analysis.

When analysed by innovation group, implementation frameworks of interventions most comprehensively covered the factors influencing implementation. Seventeen of the 22 intervention implementation frameworks (77%) included either a range of factors with some justification for inclusion (++) or comprehensive justification (+++); 16 of the 22 intervention implementation frameworks (73%) included factor descriptions (^^) and/or with relationships or operationalization (^^^). On the other hand, frameworks for implementation programs covered both the degree and depth of implementation strategies and evaluations, but were less detailed on factors. Frameworks for the implementation of guidelines, knowledge, and EBP model had lower levels of inclusion of evaluations, but were more comprehensive on strategies. Over a quarter of frameworks did not include any evaluations (13 of 49 frameworks).

## Discussion

Not surprisingly, and possibly due to a discipline effect, variations exist between both implementation frameworks for different healthcare innovations and implementation frameworks for the same healthcare innovation. The literature review sought to determine if frameworks varied depending on the innovation they were targeting. What is evident is that differences exist across frameworks regardless of whether frameworks are classified by a definition of the innovation or by terminology used to describe the innovation in the article. As such, the selection process for which framework(s) to use for a particular implementation program or study should not only be based on the type of innovation, but consider other aspects of the framework’s orientation as well as the degree of inclusion and depth of analysis of all implementation concepts.

Frameworks for particular innovations existed largely within particular settings, targeting certain users, and were often of a similar framework ‘type’, that is, they are often still tacitly discipline-specific. A disadvantage of this specificity is that end-users of the implementation framework may inadvertently follow the framework configuration of their discipline and/or innovation in which they are interested, potentially missing concepts from other implementation frameworks. As an example, if a care worker was considering to implement a guideline within their community practice and found implementation frameworks for guidelines, the frameworks would be primarily directed towards nurses, health administrators, and researchers working in hospitals or clinical settings. However, a framework for a prevention program (classified as intervention) may be more appropriate, as these are often in community settings and therefore would address influencing factors more comparable to their setting. Alternatively, a combination of frameworks may be required to cover the depth of each element.

In terms of framework ‘type’ overall there was a lack of predictive and prescriptive frameworks, which may indicate the relatively early stage of development for the implementation and knowledge translation fields. As implementation science develops one would expect that new implementation studies should lead to the development and testing of predictive framework hypotheses.

Frameworks differed in their depiction and inclusion of implementation stages. Each stage along the implementation continuum has been studied and many stages have their own frameworks, such as frameworks for diffusion up to the point of adoption or sustainability frameworks [6,40-42]. It is therefore not surprising that the pre-implementation stages (development and communication) were included less frequently; this probably reflects that adoption is often considered to be a separate field of study. Interestingly, this was particularly prevalent in the frameworks for implementation of guidelines and knowledge. Recently, a further sustainability framework has been published, which expands on the idea of adaptation and innovation improvement as being central [78].

Categorisation and focus of implementation domains also varied widely across frameworks. It appears reasonable that the nomenclature and categorisation of the domains are not critical, but rather it is important that elements at a range of levels are considered for successful implementation and sustainability to ensue. For example, in hospital settings, the organisation in some occasions was further divided to include a team or unit domain [64], and particular end-users may prefer for patients to be separate from the local environment domain [52]. Frameworks for EBP model and knowledge were particularly low on the outer settings, both the local environment and external system domains, and may benefit from investigating elements from other frameworks in future implementation efforts.

There was a limited degree of inclusion and depth of analysis across the three elements in the frameworks reviewed. It was observed that implementation frameworks for particular innovations focused more on a particular element.

When implementing an innovation, there are core concepts requiring attention. When selecting a framework to implement an innovation the user should ensure all concepts are covered or alternatively they could select a range of frameworks. In other words, concepts that should be considered include those relating to the process of implementation (the stages and steps), the innovation to be implemented, the context in which the implementation is to occur (divided into various numbers of domains), influencing factors, strategies, and evaluations. As an example, it stands to reason that an organisation wanting to implement a program may desire a more prescriptive framework that spans all the implementation stages as well as being particularly detailed on strategies and evaluations. As such, a packaged implementation program for innovations, e.g., the Replication Effective Programs Framework, may be an option [76]. On the other hand, a researcher conducting an implementation study may be wanting to focus primarily on concept of implementation, e.g., on the factors affecting implementation, and therefore the Consolidated Framework for Implementation Research would be suitable [13].

It should be noted that many implementation frameworks, models, and theories, including a number in this review, are not created to be holistic, but rather target a specific implementation concept, such as a stage, or either the factors, strategies, or evaluations. Consequently, if an all-inclusive implementation framework is desired, an alternative to selecting a comprehensive, holistic innovation-specific framework might be to choose a combination of frameworks to cover the depth of each element. This would be done by looking for framework(s) that include a range of each element the user is interested in with at least some justification for inclusion (++) or comprehensive justification (+++), plus descriptions (^^) or with relationships or operationalization (^^^).

### Emergence of a generic implementation framework

**Figure 2:**
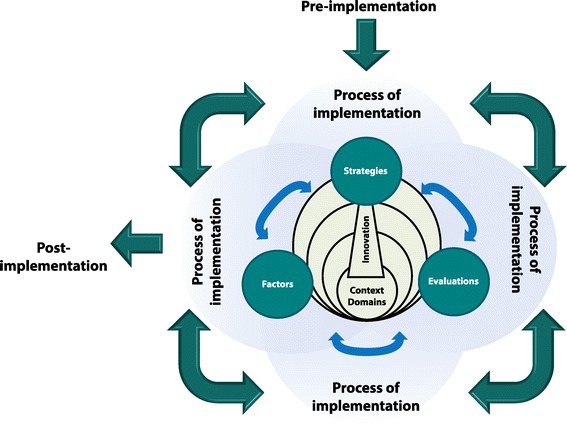
**Generic Implementation Framework (GIF).**

As implementation frameworks vary, it is valuable for researchers, policymakers, health administrators, and practitioners to have guidance of the basic components required for their implementation efforts. Across the multiple frameworks, core implementation concepts have emerged and detailed models of variables within these concepts explored; however, there seems no simple high level illustration of these overarching concepts. Furthermore, as many frameworks are not holistic, by design or otherwise, without knowledge or illustration of the core implementation concepts that should be considered, it is difficult to determine if a single or multiple meta-frameworks or models are required. A Generic implementation framework (GIF) has been proposed to depict the core concepts of implementation (Figure 2). Foremost to implementation is the non-linear and recursive nature of the implementation process (illustrated by the double arrows and overlapping circles). This process is then able to be divided into a series of stages and/or steps. At the centre of the framework is the innovation to be implemented and surrounding the innovation are the contextual domains or levels of influence. Throughout the implementation process, at each stage and for each domain, there are factors, strategies, and evaluations that will influence the course of implementation and should be considered. It is important to note that the GIF is not a new framework but rather a composite of what is represented in most, if not all, other frameworks. Using the GIF as a starting point or checklist ensures the framework(s) chosen cover the core implementation concepts.

The GIF may be used as a memory aid, to ensure that, when an implementation effort (investigation, protocol, or program) is being designed, all concepts are considered and that the selection of framework(s) sufficiently covers them all. In this way, the GIF may be utilised as a base for the development of implementation protocols or programs and then tailored for use, depending of the innovation, user, setting, discipline, and objective. Meta-frameworks, models, or theories and particular discipline, innovation, or setting-specific variables may be added accordingly to each concept. The framework analysis may assist a user to choose an appropriate framework or combination of frameworks for their particular study or project. This may be done by using the table of analysis as a decision-support tool, whereby the end-user factors in their circumstances and objectives and compares this to the frameworks’ targeted innovation, setting, type, and aim, along with the stages and domains it addresses and the degree and depth in which the elements are covered. For instance, guideline implementation frameworks, were essentially descriptive, based on clinical settings, were largely focussed on two domains, the innovation (the characteristics of the guideline to be implemented) and individuals, often did not include the stages of exploration and installation, and lacked comprehensiveness (degree and depth) of the evaluations element. Therefore, following on with the previous example of a care worker considering the implementation of a clinical guideline within their community practice, they may benefit from looking outside of the guideline implementation literature to frameworks for implementation programs which cover the missing stages and evaluations element to a greater extent. Furthermore, combining such a framework with a prevention program framework to address the factors associated with the user’s orientation as a care worker in the community could be considered.

### Limitations

Studies applying frameworks were not included unless a new framework was proposed. As such, further details to constructs may have been added to a framework, but not included in the review. Similarly, only the original reference per framework was included, unless subsequent changes were made to the framework, even if the depth of analysis was expanded in later papers. These exclusions could have affected the degree and depth of analysis; however, this has not affected the explanation of the framework or the resulting GIF. Moreover, it means that influential frameworks within implementation science published prior to 2004 are omitted, but these have been analysed in previously published literature reviews [79-81].

Classifying innovations based on definitions would have impacted the groupings and overall analysis (for example, by definition, clinical guidelines are used to implement evidence based practices and therefore could be classified as a health intervention rather than have their own group). This could be seen as a limitation, but it does not reduce the validity of the results, as the objective of analysis was to determine if an end-user was choosing a framework for a particular innovation would the frameworks targeting this innovation be the most suitable, or would a framework, created for the implementation of a different innovation, add further details on implementation concepts.

Finally, both the article inclusion and data extraction was performed by a single reviewer (JCM), with assistance from a secondary member when doubts arose (SIB). This may have influenced the coding of comprehensiveness of the frameworks (if different reviewers’ were to have arrived at different classifications of the evaluative components degree and depth); however, the definitions for data extraction were developed to minimise uncertainties.

## Conclusions

The literature review revealed variations in implementation frameworks of innovations in healthcare. Core concepts of implementation should be considered for every implementation effort, yet differences were seen in the structure and order in which the implementation process and domains were depicted, as well as the comprehensiveness of factors, strategies, and evaluations. Concepts that should be considered for successful implementation include those relating to the process of implementation (the stages and steps), the innovation to be implemented, the context in which the implementation is to occur (divided into various numbers of domains), influencing factors, strategies, and evaluations. The GIF was developed to ensure chosen frameworks, meta-frameworks, models, or theories as well as particular discipline, innovation, or setting-specific variables, cover the core implementation concepts.

## Additional files

Additional file 1:
**Definitions.**
Additional file 2:
**Table of analysis.**

## Abbreviations

EBP: Evidence-based practice
GIF: Generic implementation framework
