# Supplementary material for: A systematic review of implementation frameworks of innovations in healthcare and resulting generic implementation framework
Source: Health Res Policy Syst. 2015 Mar 14;13:16. doi: 10.1186/s12961-015-0005-z (PMC4364490; doi:10.1186/s12961-015-0005-z)
Supplement: Additional file 2: — Table of analysis. [file 12961_2015_5_MOESM2_ESM.pdf]

Additional File 2: Framework analysis

Innovation category	Framework		Orientation				Type	Stages						Domains					Elements		
	**Title**	**Reference**	**Innovation**	**Setting**	**User(s)**	**Aim**	**Descriptive Explanatory Predictive**	**Development**	**Communication**	**Exploration**	**Installation**	**Operation**	**Sustainability**	**Innovation**	**Individuals**	**Organisation**	**Local environment**	**External system**	**Factors**	**Strategies**	**Evaluations**
Interventions	Conceptual framework for sustainability of public health programs	Scheirer MA & Dearing JW. 2011[1]	Funded programs of healthcare, health promotion & disease prevention interventions	Health organisations and community settings	Researchers and funders	Provide guidance, for research and evaluation, of health program sustainability	Predictive						✓	✓		✓	✓	✓	++<br>^^	+<br>^	+++<br>^^
Interventions	Sustainability planning model	Johnson K et al. 2004[2]	Substance abuse prevention innovations, programs and strategies	Organisational, community, state or national level substance abuse prevention systems	Researchers and practitioners	Highlight the key factors relating to sustaining innovations and how to address them	Prescriptive Explanatory						✓	✓		✓			++<br>^^^	++<br>^^	++<br>^^
Interventions	Capacity for sustainability framework	Schell SF et al. 2013[3]	Public health programs (prevention)	Community practice settings	Researchers, funders and practitioners	Aid public health programs in conceptualizing their capacity for sustainability	Descriptive						✓	✓		✓	✓	✓	++<br>^	+<br>^	+<br>^
Interventions	Interactive Systems Framework for dissemination and implementation (ISF)	Wandersman A et al. 2008[4]	Prevention innovations (programs, policies, processes & principles)	Organisational, community, state or national level	Multiple stakeholders (e.g. funders, practitioners, researchers)	Heuristic to help clarify the issues related to how to move what is known about prevention into widespread use	Descriptive Explanatory		✓	✓	✓	✓			✓	✓	✓	✓	+<br>^	++<br>^^	nil

Innovation category	Framework		Orientation				Type	Stages						Domains					Elements		
	Title	Reference	Innovation	Setting	User(s)	Aim	Descriptive<br>Explanatory<br>Predictive	Development	Communication	Exploration	Installation	Operation	Sustainability	Innovation	Individuals	Organisation	Local environment	External system	Factors	Strategies	Evaluations
Interventions	TCU Program Change Model	Simpson DD & Flynn PM. 2007[5]	Community drug treatment programs and health service innovations	Community-based organisations	Researchers	Organize and summarize implementation results	Descriptive<br>Explanatory			✓	✓	✓	✓	✓	✓	✓			++<br>^^^	++<br>^^	++<br>^^^
Interventions	Ecological framework for understanding effective implementation	Durlak JA & DuPre EP. 2008[6]	Promotion and prevention programs/ interventions	Community settings	Researchers	Framework to depict the factors that affect implementation	Explanatory<br>Predictive		✓	✓	✓	✓	✓	✓	✓	✓	✓		+++<br>^^	++<br>^^	++<br>^^
Interventions	Core implementation components	Fixsen DL et al. 2009[7]	Research-based prevention and treatment services, interventions and programs	Human service settings	Researchers and practitioners	Present the critical links of implementation stages and core components in the science to service chain	Descriptive			✓	✓	✓	✓		✓	✓		✓	++<br>^	+++<br>^^	+<br>^
Interventions	Model of sustaining innovations in their effectiveness	Racine DP. 2006[8]	Health and human service innovations	Human and health services	Creators, funders, adopters, researchers	Describe factors that appear to play important roles in whether innovations are replicated and sustained in their effectiveness	Explanatory<br>Predictive			✓		✓	✓	✓		✓	✓	✓	++<br>^^^	++<br>^^	nil
Interventions	Framework of dissemination in health services intervention research	Mendel P et al. 2008[9]	Evidence-based health interventions (mental health)	Community healthcare settings	Health services researchers	Evaluation and capacity focus (stakeholder/ community partnerships)	Descriptive<br>Explanatory				✓	✓	✓		✓	✓	✓	✓	+<br>^^^	+<br>^	++<br>^^^

Innovation category	Framework		Orientation				Type	Stages						Domains					Elements		
	**Title**	**Reference**	**Innovation**	**Setting**	**User(s)**	**Aim**	**Descriptive Explanatory Predictive**	**Development**	**Communication**	**Exploration**	**Installation**	**Operation**	**Sustainability**	**Innovation**	**Individuals**	**Organisation**	**Local environment**	**External system**	**Factors**	**Strategies**	**Evaluations**
Interventions	Conceptual model of implementation phases and factors affecting implementation in public service sectors	Aarons GA, Hurlburt M and McCue Horwitz S. 2011[10]	Evidence-based practices (mental health services and human service technologies)	Public sector systems serving children and families (child welfare)	Multiple stakeholders	Framework which includes the importance of variables at each implementation phase	Descriptive Explanatory			✓	✓	✓	✓	✓	✓	✓	✓	✓	++<br>^^^	+<br>^	nil
Interventions	Integrated 2-phase TCU approach to strategic system change	Lehman WEK et al. 2011[11]	Evidence-based practices (technologies)	Substance abuse treatment services	Researchers and organisations	Present a biphasic framework for innovation planning and implementation	Explanatory	✓		✓	✓	✓	✓	✓	✓	✓			++<br>^^	++<br>^^	+++<br>^^^
Interventions	ATTC Network model of technology transfer in the innovation process	Addiction Technology Transfer Center Network Technology Transfer Workgroup. 2011[12]	Evidence based practices (technologies)	Substance abuse treatment organisations	Stakeholders (funders, organisations educational institutions, practitioners) and researchers	Field-driven conceptual model of innovation process and to provide a comprehensive taxonomy	Descriptive	✓	✓	✓		✓		✓	✓	✓			+<br>^	+<br>^	nil
Interventions	Practical, Robust Implementation and Sustainability Model (PRISM)	Feldstein AC & Glasgow RE. 2008[13]	Health interventions (programs, technologies, evidence-based practices)	Healthcare practices	Researchers and healthcare managers	Integrate key features of program design, predictors of implementation and diffusion, and appropriate outcomes measures	Descriptive Explanatory				✓	✓	✓	✓	✓	✓	✓	✓	++<br>^^^	++<br>^^^	++<br>^

Innovation category	Framework		Orientation				Type	Stages						Domains					Elements		
	Title	Reference	Innovation	Setting	User(s)	Aim	Descriptive Explanatory Predictive	Development	Communication	Exploration	Installation	Operation	Sustainability	Innovation	Individuals	Organisation	Local environment	External system	Factors	Strategies	Evaluations
Interventions	Consolidated Framework of Implementation Research (CFIR)	Damschorder LJ et al. 2009[14]	Health interventions	Multiple contexts	Health services researchers	Consolidate all factors that may be encountered, promote theory development and guide formative evaluations	Descriptive		✓		✓	✓		✓	✓	✓	✓	✓	+++ ^^^	+ ^^	+ ^^^
Interventions	A model of diffusion in service organizations	Greenhalgh T et al. 2004[15]	Health service innovations	Healthcare service organisations	Researchers	Depict how to spread and sustain innovations in health service delivery and organisation	Descriptive Explanatory	✓	✓	✓		✓	✓	✓	✓	✓	✓	✓	+++ ^^	++ ^^	nil
Interventions	Factors that determine the rate of adopting of innovations from research into practice	Bradley EH et al. 2004[16]	Clinical programs and innovations	Multiple settings. Hospital, nursing home, paediatrics, pharmacy	Researchers	Describe the factors and steps learned about diffusing innovations into practice	Descriptive Explanatory					✓	✓	✓		✓		✓	++ ^^	++ ^^	+ ^
Interventions	Conceptual framework of complex innovation implementation	Helfrich CD et al. 2007[17]	Complex innovations	Healthcare organisations	Researchers, managers and practitioners	Adapt an organisational framework of innovation implementation for utility in healthcare	Predictive					✓				✓			++ ^^^	+ ^^^	+ ^^^
Interventions	Normalisation Process Theory (NPT)	May C & Finch T. 2009[18]	Complex practices: Business processes or complex interventions	Organisations or institutions	Researchers	Propose a working model of implementation, embedding and integration	Descriptive Explanatory Predictive				✓	✓	✓	✓	✓	✓	✓		++ ^^	++ ^^	++ ^^

Innovation category	Framework		Orientation				Type	Stages						Domains					Elements		
	Title	Reference	Innovation	Setting	User(s)	Aim	Descriptive<br>Explanatory<br>Predictive	Development	Communication	Exploration	Installation	Operation	Sustainability	Innovation	Individuals	Organisation	Local environment	External system	Factors	Strategies	Evaluations
Interventions	Normalisation Process Theory (NPT)	May C. 2013[19]	Innovations: Shared decision making in doctor and nurse-patient interactions	Healthcare organisations	Researchers	Specify specific set of activities involved in implementation (process) and embedding (state) of innovations	Descriptive<br>Explanatory<br>Predictive				✓	✓	✓						nil	++<br>^^	++<br>^
Interventions	General theory of implementation	May C. 2013[20]	Complex interventions	Clinical settings	Researchers and practitioners	Foundation for understanding, designing, predicting, and evaluating dynamic implementation processes	Descriptive<br>Explanatory<br>Predictive					✓		✓	✓	✓	✓	✓	++<br>^^	+<br>^^^	nil
Interventions	Model for success and breakdown factors of shared governance	Ballard N. 2010[21]	Shared governance	Practice settings	Nursing leaders	Summarise human and structural factors that contribute to success or breakdown of shared governance practice models	Descriptive				✓	✓	✓		✓	✓	✓		+<br>^	++<br>^^	nil
Interventions	Model and checklist for telehealth	Joseph V et al. 2011[22]	Telehealth	Healthcare organisations	Practitioners	Identify the key challenges for telehealth projects (trials) & produce a model and checklist to ensure success	Descriptive				✓	✓	✓		✓	✓	✓		++<br>^^	++<br>^^	+<br>^
Guidelines	Three phase implementation model	Carey M, Buchnan H & Sanson-Fisher R. 2009[23]	Evidence-based guideline recommenda-tions	Healthcare	Guideline developers	Provide strategies targeting factors associated with behavioural change in a logical sequence	Descriptive	✓	✓		✓	✓		✓	✓	✓	✓		+<br>^	++<br>^^^	+<br>^

Innovation category	Framework		Orientation				Type	Stages						Domains					Elements		
	Title	Reference	Innovation	Setting	User(s)	Aim	Descriptive Explanatory Predictive	Development	Communication	Exploration	Installation	Operation	Sustainability	Innovation	Individuals	Organisation	Local environment	External system	Factors	Strategies	Evaluations
Guidelines	Contingency model of innovation adoption	Berta W et al. 2005[24]	Clinical practice guidelines to improve quality of care	Long-term care facilities	Researchers	Describe factors that contribute to capacity building at each stage of innovation adoption	Predictive		✓			✓	✓	✓	✓	✓	✓	✓	++ ^^	+ ^	nil
Guidelines	Model matrix of factors in implementation of practice change	Hader JM et al. 2007[25]	Clinical practice guidelines	Doctors in clinical practice	Guideline developers	Insights into the determinants of change which may help identify means to increase successful implementation	Descriptive		✓	✓		✓	✓	✓	✓			✓	++ ^^	+ ^	nil
Guidelines	Guideline implementability framework	Gagliardi AR et al. 2011[26]	Guidelines	Healthcare	Guideline developers	Identify and define features that facilitate guideline use	Descriptive					✓		✓					+ ^	+ ^	+ ^
Knowledge	10-step model for inducing change in professional behaviour	Grol R & Wensing M. 2004[27]	Evidence (Change in practice)	Health services	Researchers	How to identify, categorize and use barriers and incentives to change practice, to tailor interventions and to facilitate desired change	Prescriptive		✓	✓		✓	✓	✓	✓	✓		✓	++ ^^	++ ^	nil
Knowledge	John Hopkins Quality and Safety Research Group translating evidence into practice model	Pronovost P, Berenholtz S & Needham D. 2008[28]	Evidence (evidence-based therapies & interventions)	Hospitals - clinical practice	Researchers and clinicians	Embed a method of knowledge translation in a collaborative team model	Prescriptive	✓			✓	✓	✓						+ ^	+ ^^	+ ^^
Knowledge	Promoting Action on Research Implementation in Health Services (PARIHS)	Kitson AL et al. 2008[29]	Evidence	Hospital	Researchers and practitioners	Heuristic to frame research or knowledge translation endeavours as two stage diagnostic and evaluative approach	Explanatory				✓	✓		✓	✓	✓			++ ^^^	++ ^^^	++ ^^

Innovation category	Framework		Orientation				Type	Stages						Domains					Elements		
	Title	Reference	Innovation	Setting	User(s)	Aim	Descriptive<br>Explanatory<br>Predictive	Development	Communication	Exploration	Installation	Operation	Sustainability	Innovation	Individuals	Organisation	Local environment	External system	Factors	Strategies	Evaluations
Knowledge	Revised PARIHS framework for a task-orientated approach to implementation	Stetler CB et al. 2011[30]	Evidence based practices	Veterans Health Administration healthcare settings or similar	Researchers	Revising PARIHS framework for use in task-orientated implementation	Explanatory				✓	✓		✓	✓	✓			++<br>^^^	+++<br>^^^	+++<br>^^^
Knowledge	Promoting Action on Research Implementation in Health Services (PARIHS)	Rycroft-Malone J et al. 2013[31]	Evidence	Hospital	Researchers and practitioners	Represent the process of implementation of evidence to practice	Explanatory				✓	✓		✓	✓	✓			++<br>^^	++<br>^^^	++<br>^
Knowledge	Critical Realism & the Arts Research Utilization Model (CRARUM)	Kontos PC & Poland BD. 2009[32]	Knowledge	Healthcare settings	Researchers	Incorporate the interpretive dimension of evidence and complexity of healthcare settings into a Knowledge Translation model	Descriptive<br>Explanatory			✓	✓	✓	✓	✓	✓	✓	✓		++<br>^^	++<br>^^^	++<br>^^
Knowledge	Analytic framework: moving knowledge into action	Best A, Hiatt RA & Norman CD. 2008[33]	Cancer control strategies (knowledge)	Cancer control systems	Practitioners	Integrate knowledge into the system (decisions, practices and policies) with communication as a central role	Descriptive		✓	✓		✓		✓	✓	✓	✓	✓	+<br>^	++<br>^^	nil
Knowledge	Knowledge to Action (KTA)	Graham ID et al. 2006[34]	Knowledge (research)	Healthcare practice	Healthcare practitioners, policymakers, patients, and the public	Conceptual map of the Knowledge-To-Action process	Prescriptive	✓	✓	✓	✓	✓	✓	✓	✓	✓			+<br>^	+<br>^	++<br>^^^
Knowledge	Sticky knowledge	Elwyn G, Taubert M & Kowalczuk J. 2007[35]	Knowledge (evidence)	Primary care	Clinicians and management	Demonstrate sticky knowledge may play a role in helping overcome barriers to transfer	Descriptive			✓	✓	✓	✓	✓	✓	✓			++<br>^^	+<br>^	nil

Innovation category	Framework		Orientation				Type	Stages						Domains					Elements		
	Title	Reference	Innovation	Setting	User(s)	Aim	Descriptive<br>Explanatory<br>Predictive	Development	Communication	Exploration	Installation	Operation	Sustainability	Innovation	Individuals	Organisation	Local environment	External system	Factors	Strategies	Evaluations
Knowledge	Joint Venture Model of Knowledge Utilization (JVMKU)	Edgar L et al. 2006[36]	Knowledge	Nursing and healthcare	Nurses	Practical guide for critical reflection on issues affecting the process of knowledge utilisation	Descriptive<br>Explanatory		✓			✓		✓	✓	✓	✓	✓	++<br>^	++<br>^	+<br>^
Knowledge	Knowledge-value chain	Landry R et al. 2006[37]	Knowledge (research)	Public Health organisations (e.g. WHO)		Use of knowledge management for the capability to acquire, create, share and apply knowledge	Descriptive	✓	✓			✓		✓		✓			+<br>^	+<br>^	+<br>^
Knowledge	Multisystem model of knowledge integration and translation	Palmer D & Kramlich D. 2011[38]	Knowledge	Healthcare	Nurse researchers	Generation, integration and implementation of knowledge into practice based on concepts of communities of practice	Descriptive<br>Explanatory<br>Prescriptive	✓	✓	✓	✓	✓	✓		✓	✓	✓		+<br>^	++<br>^^^	+<br>^
Knowledge	Knowledge Use in Pain Care (KUPC)	Latimer MA, Ritchie JA & Johnston CC. 2010[39]	Knowledge (pain care for children)	Nursing practice in hospitals	Healthcare administrators clinical leaders, researchers	Link theory and evidence from clinical and administrative perspectives to implementation activities	Descriptive					✓			✓	✓	✓	✓	++<br>^^	++<br>^^	nil
Knowledge	Collaborative model of knowledge translation in clinical Settings	Baumbusch JL et al. 2008[40]	Research findings	Clinical settings		Provide an interactive model of knowledge translation	Explanatory	✓	✓	✓	✓	✓	✓	✓			✓	✓	++<br>^	+<br>^^	+<br>^
Knowledge	Knowledge integration model	Gauthier N et al. 2005[41]	Knowledge/ evidence	Clinical settings	Researchers, clinicians and decision makers	Illustrate the dynamic and reiterative nature of knowledge integration supported by on-going dialogue	Descriptive	✓	✓	✓		✓		✓		✓	✓		+<br>^	++<br>^^	+<br>^

Innovation category	Framework		Orientation				Type	Stages						Domains					Elements		
	Title	Reference	Innovation	Setting	User(s)	Aim	Descriptive<br>Explanatory<br>Predictive	Development	Communication	Exploration	Installation	Operation	Sustainability	Innovation	Individuals	Organisation	Local environment	External system	Factors	Strategies	Evaluations
Evidence based practice	Trinity model of evidence-based practice	Vratny A & Shriver D. 2007[42]	Evidence based practice model of care	Medical centre (hospital)	Nursing administrators	Use a tree metaphor to depict the core components to overcome barriers and encourage EBP implementation	Descriptive				✓	✓		✓	✓	✓	✓	✓	++<br>^^	++<br>^^^	++<br>^^
Evidence based practice	Process for the use of evidence-based practice model for staff nurses	Reavy K & Tavernier S. 2008[43]	Evidence based practice model	Medical centre (hospital)	Staff nurses	Staff nurses to recognize ownership of their practice and role in changing practice	Prescriptive	✓		✓	✓	✓	✓		✓				+<br>^	++<br>^^	+<br>^^
Evidence based practice	Tyler collaborative model for evidence-based practice	Olade RA. 2004[44]	Evidence based practice	Healthcare settings	Nurses and other healthcare professionals	Guide collaborative efforts toward evidence-based nursing practice	Prescriptive			✓	✓	✓	✓		✓	✓			++<br>^	++<br>^^	+<br>^^
Evidence based practice	Steps in building an EBP Program	MacRobert M. 2008[45]	Evidence based practice	Case management practice setting	Case managers	Describe steps and strategies to move EBP into practice	Explanatory<br>Prescriptive				✓	✓		✓	✓	✓			+<br>^	++<br>^^^	++<br>^
Evidence based practice	Advancing Research and Clinical Practice Through Close Collaboration Model (ARCC)	Melnyk BM et al. 2010[46]	Evidence based practice	Hospitals and healthcare systems	EBP implementers	Guide system-wide implementation and sustainability of EBP to improve quality of care and patient outcomes	Predictive				✓	✓			✓	✓			+<br>^^	++<br>^^	+<br>^^

Innovation category	Framework		Orientation				Type	Stages						Domains					Elements		
	**Title**	**Reference**	**Innovation**	**Setting**	**User(s)**	**Aim**	**Descriptive Explanatory Predictive**	**Development**	**Communication**	**Exploration**	**Installation**	**Operation**	**Sustainability**	**Innovation**	**Individuals**	**Organisation**	**Local environment**	**External system**	**Factors**	**Strategies**	**Evaluations**
Implementation programs	Replicating Effective Programs Framework (REP)	Kilbourne AM et al. 2007[47]	Program for implementing clinical and health service interventions	Community-based organisations	Researchers, intervention developers, stakeholders	Framework that can be applied to implement interventions	Descriptive Prescriptive	✓	✓	✓	✓	✓	✓	✓	✓	✓	✓	✓	++ ^	+++ ^^^	++ ^^
Implementation programs	CHANGE model (customised, holistic, analytical, network-building, grassroots, evaluatory)	Vega MY. 2009[48]	Program for implementing packaged prevention interventions	Community-based organisations	Practitioners and researchers	Approach to provide skills to implement and capacity to reorient to interventions	Descriptive Prescriptive				✓	✓	✓	✓	✓	✓	✓	✓	+ ^^	++ ^^^	++ ^^
Implementation programs	ARC organisational and community intervention model	Glisson C & Schoenwald SK 2005[49]	Program for implementing social and mental health treatments and services for children	Isolated community practice settings	Practitioners and researchers	Address the 'fit' between the social context and service technology, address technical, social and strategic factors.	Descriptive Explanatory				✓	✓	✓	✓	✓	✓	✓		+ ^^	++ ^^^	++ ^^

+ The framework itemizes a limited range of factors, strategies or evaluations with no explanation for their inclusion
++ The framework itemizes a range of factors, strategies or evaluations with some justification for their inclusion
+++ The framework itemizes a comprehensive range of factors, strategies or evaluations based on a literature review or expert consensus
^ Factors, strategies or evaluations provided as a list without description
^^ Factors, strategies or evaluations provided with definitions
^^^ Factors, strategies or evaluations provided with explanations which include the relationships between or within the elements (factors, strategies and evaluations) or mechanisms for operationalization

EBP = Evidence based practice model of practice; EBPs – Evidence-based practices; TCU – Texas Christian University

References

1. Scheirer MA, Dearing JW: **An agenda for research on the sustainability of public health programs.** *Am J Public Health* 2011, **101:**2059-2067.
2. Johnson K, Hays C, Center H, Daley C: **Building capacity and sustainable prevention innovations: a sustainability planning model.** *Eval Program Plann* 2004, **27:**135-149.
3. Schell S, Luke D, Schooley M, Elliott M, Herbers S, Mueller N, Bunger A: **Public health program capacity for sustainability: a new framework.** *Implement Sci* 2013, **8:**15.
4. Wandersman A, Duffy J, Flaspohler P, Noonan R, Lubell K, Stillman L, Blachman M, Dunville R, Saul J: **Bridging the gap between prevention research and practice: the interactive systems framework for dissemination and implementation.** *Am J Community Psychol* 2008, **41:**171-181.
5. Simpson DD, Flynn PM: **Moving innovations into treatment: a stage-based approach to program change.** *J Subst Abuse Treat* 2007, **33:**111-120.
6. Durlak JA, DuPre EP: **Implementation matters: a review of research on the influence of implementation on program outcomes and the factors affecting implementation.** *Am J Community Psychol* 2008, **41:**327-350.
7. Fixsen DL, Blase KA, Naoom SF, Wallace F: **Core implementation components.** *Res Soc Work Pract* 2009, **19:**531-540.
8. Racine DP: **Reliable effectiveness: a theory on sustaining and replicating worthwhile innovations.** *Adm Policy Ment Health* 2006, **33:**356-387.
9. Mendel P, Meredith LS, Schoenbaum M, Sherbourne CD, Wells KB: **Interventions in organizational and community context: a framework for building evidence on dissemination and implementation in health services research.** *Adm Policy Ment Health* 2008, **35:**21-37.
10. Aarons G, Hurlburt M, Horwitz S: **Advancing a conceptual model of evidence-based practice implementation in public service sectors.** *Adm Policy Ment Health* 2011, **38:**4-23.
11. Lehman WE, Simpson DD, Knight DK, Flynn PM: **Integration of treatment innovation planning and implementation: strategic process models and organizational challenges.** *Psychol Addict Behav* 2011, **25:**252.
12. Addiction Technology Transfer Center (ATTC) Network Technology Transfer Workgroup: **Research to practice in addiction treatment: key terms and a field-driven model of technology transfer.** *J Subst Abuse Treat* 2011, **41:**169-178.
13. Feldstein AC, Glasgow RE: **A practical, robust implementation and sustainability model (PRISM).** *Jt Comm J Qual Patient Saf* 2008, **34:**228-243.
14. Damschroder LJ, Aron DC, Keith RE, Kirsh SR, Alexander JA, Lowery JC: **Fostering implementation of health services research findings into practice: a consolidated framework for advancing implementation science.** *Implement Sci* 2009, **4:**50-50.
15. Greenhalgh T, Robert G, Macfarlane F, Bate P, Kyriakidou O: **Diffusion of innovations in service organizations: systematic review and recommendations.** *Milbank Q* 2004, **82:**581-629.
16. Bradley EH, Fund C: **Translating research into practice: speeding the adoption of innovative health care programs.** *Commonw Fund* 2004, **724:**1-12.
17. Helfrich CD, Weiner BJ, McKinney MM, Minasian L: **Determinants of implementation effectiveness.** *Med Care Res Rev* 2007, **64:**279-303.
18. May C, Finch T: **Implementing, embedding, and integrating practices: an outline of normalization process theory.** *Sociology* 2009, **43:**535-554.
19. May C: **Agency and implementation: understanding the embedding of healthcare innovations in practice.** *Soc Sci Med* 2013, **78:**26-33.
20. May C: **Towards a general theory of implementation.** *Implement Sci* 2013, **8:**18.

21. Ballard N: **Factors associated with success and breakdown of shared governance.** *J Nurs Adm* 2010, **40:**411-416.
22. Joseph V, West RM, Shickle D, Keen J, Clamp S: **Key challenges in the development and implementation of telehealth projects.** *J Telemed Telecare* 2011, **17:**71-77.
23. Carey M, Buchan H, Sanson-Fisher R: **The cycle of change: implementing best-evidence clinical practice.** *Int J Qual Health Care* 2009, **21:**37-43.
24. Berta W, Teare GF, Gilbart E, Ginsburg LS, Lemieux-Charles L, Davis D, Rappolt S: **The contingencies of organizational learning in long-term care: factors that affect innovation adoption.** *Health Care Manage Rev* 2005, **30:**282-292.
25. Hader JM, White R, Lewis S, Foreman JL, McDonald PW, Thompson LG: **Doctors' views of clinical practice guidelines: a qualitative exploration using innovation theory.** *J Eval Clin Pract* 2007, **13:**601-606.
26. Gagliardi AR, Brouwers MC, Palda VA, Lemieux-Charles L, Grimshaw JM: **How can we improve guideline use? A conceptual framework of implementability.** *Implement Sci* 2011, **6:**26.
27. Grol R, Wensing M: **What drives change? Barriers to and incentives for achieving evidence-based practice.** *Med J Aust* 2004, **180:**S57.
28. Pronovost PJ, Berenholtz SM, Needham DM: **Translating evidence into practice: a model for large scale knowledge translation.** *BMJ* 2008, **337:**963-965.
29. Kitson AL, Rycroft-Malone J, Harvey G, McCormack B, Seers K, Titchen A: **Evaluating the successful implementation of evidence into practice using the PARiHS framework: theoretical and practical challenges.** *Implement Sci* 2008, **3:**1-1.
30. Stetler CB, Damschroder LJ, Helfrich CD, Hagedorn HJ: **A Guide for applying a revised version of the PARIHS framework for implementation.** *Implement Sci* 2011, **6:**99-99.
31. Rycroft-Malone J, Seers K, Chandler J, Hawkes C, Crichton N, Allen C, Bullock I, Strunin L: **The role of evidence, context, and facilitation in an implementation trial: implications for the development of the PARIHS framework.** *Implement Sci* 2013, **8:**28.
32. Kontos PC, Poland BD: **Mapping new theoretical and methodological terrain for knowledge translation: contributions from critical realism and the arts.** *Implement Sci* 2009, **4:**1.
33. Best A, Hiatt RA, Norman CD: **Knowledge integration: conceptualizing communications in cancer control systems.** *Patient Educ Couns* 2008, **71:**319-327.
34. Graham ID, Logan J, Harrison MB, Straus SE, Tetroe J, Caswell W, Robinson N: **Lost in knowledge translation: Time for a map?** *J Contin Educ Health Prof* 2006, **26:**13-24.
35. Elwyn G, Taubert M, Kowalczuk J: **Sticky knowledge: a possible model for investigating implementation in healthcare contexts.** *Implement Sci* 2007, **2:**44.
36. Edgar L, Herbert R, Lambert S, MacDonald JA, Dubois S, Latimer M: **The joint venture model of knowledge utilization: a guide for change in nursing.** *Nurs Leadersh (Tor Ont)* 2006, **19:**41-55.
37. Landry R, Amara N, Pablos-Mendes A, Shademani R, Gold I: **The knowledge-value chain: a conceptual framework for knowledge translation in health.** *Bull World Health Organ* 2006, **84:**597-602.
38. Palmer D, Kramlich D: **An introduction to the multisystem model of knowledge integration and translation.** *ANS Adv Nurs Sci* 2011, **34:**29-38.

39. Latimer MA, Ritchie JA, Johnston CC: **Individual nurse and organizational context considerations for better knowledge use in pain care.** *J Pediatr Nurs* 2010, **25:**274-281.
40. Baumbusch JL, Kirkham SR, Khan KB, McDonald H, Semeniuk P, Tan E, Anderson JM: **Pursuing common agendas: a collaborative model for knowledge translation between research and practice in clinical settings.** *Res Nurs Health* 2008, **31:**130-140.
41. Gauthier N, Ellis K, Bol N, Stolee P: **Beyond knowledge transfer: a model of knowledge integration in a clinical setting.** *Healthc Manage Forum* 2005, **18:**33-37.
42. Vratny A, Shriver D: **A conceptual model for growing evidence-based practice.** *Nurs Adm Q* 2007, **31:**162-170.
43. Reavy K, Tavernier S: **Nurses reclaiming ownership of their practice: implementation of an evidence-based practice model and process.** *J Contin Educ Nur* 2008, **39:**166-172.
44. Olade RA: **Strategic collaborative model for evidence-based nursing practice.** *Worldviews Evid Based Nurs* 2004, **1:**60-68.
45. MacRobert M: **A leadership focus on evidence-based practice: tools for successful implementation.** *Prof Case Manag* 2008, **13:**97-101.
46. Melnyk BM, Fineout-Overholt E, Gallagher-Ford L, Stillwell SB: **Evidence-based practice, step by step: sustaining evidence-based practice through organizational policies and an innovative model.** *Am J Nurs* 2011, **111:**57-60.
47. Kilbourne AM, Neumann MS, Pincus HA, Bauer MS, Stall R: **Implementing evidence-based interventions in health care: application of the replicating effective programs framework.** *Implement Sci* 2007, **2:**42.
48. Vega MY: **The CHANGE approach to capacity-building assistance.** *AIDS Educ Prev* 2009, **21:**137-151.
49. Glisson C, Schoenwald SK: **The ARC organizational and community intervention strategy for implementing evidence-based children's mental health treatments.** *Ment Health Serv Res* 2005, **7:**243-259.
